# Isoflurane Reversibly Destabilizes Hippocampal Dendritic Spines by an Actin-Dependent Mechanism

**DOI:** 10.1371/journal.pone.0102978

**Published:** 2014-07-28

**Authors:** Jimcy Platholi, Karl F. Herold, Hugh C. Hemmings, Shelley Halpain

**Affiliations:** 1 Division of Biological Sciences, University of California San Diego and Sanford Consortium for Regenerative Medicine, San Diego, California, United States of America; 2 Departments of Anesthesiology and Pharmacology, Weill Cornell Medical College, New York, New York, United States of America; Imperial College London, Chelsea & Westminster Hospital, United Kingdom

## Abstract

General anesthetics produce a reversible coma-like state through modulation of excitatory and inhibitory synaptic transmission. Recent evidence suggests that anesthetic exposure can also lead to sustained cognitive dysfunction. However, the subcellular effects of anesthetics on the structure of established synapses are not known. We investigated effects of the widely used volatile anesthetic isoflurane on the structural stability of hippocampal dendritic spines, a postsynaptic structure critical to excitatory synaptic transmission in learning and memory. Exposure to clinical concentrations of isoflurane induced rapid and non-uniform shrinkage and loss of dendritic spines in mature cultured rat hippocampal neurons. Spine shrinkage was associated with a reduction in spine F-actin concentration. Spine loss was prevented by either jasplakinolide or cytochalasin D, drugs that prevent F-actin disassembly. Isoflurane-induced spine shrinkage and loss were reversible upon isoflurane elimination. Thus, isoflurane destabilizes spine F-actin, resulting in changes to dendritic spine morphology and number. These findings support an actin-based mechanism for isoflurane-induced alterations of synaptic structure in the hippocampus. These reversible alterations in dendritic spine structure have important implications for acute anesthetic effects on excitatory synaptic transmission and synaptic stability in the hippocampus, a locus for anesthetic-induced amnesia, and have important implications for anesthetic effects on synaptic plasticity.

## Introduction

The mechanisms underlying general anesthesia, a reversible pharmacologic coma-like state characterized by amnesia, loss of consciousness and immobility, remain poorly understood. Although no singular molecular or cellular target has been identified that can explain these diverse effects, volatile anesthetics are known to regulate synaptic transmission by modulating various ligand- and voltage-gated ion channels at synaptic and extrasynaptic sites [1], [2]. While these effects have been assumed to be fully reversible upon drug elimination, recent evidence suggests that exposure of the early postnatal or aging brain to anesthetics can induce long-lasting impairment in cognitive function and synaptic plasticity [3], [4]. Anesthetics can also interfere with neuronal polarity establishment and axon pathfinding in immature cultured mouse neurons [5]. These recent findings raise concerns that general anesthetics might lead to persistent disruption of developing or established synaptic connections and neuronal networks.

Dendritic spines are actin-rich postsynaptic structures at most excitatory synapses in mammalian brain that represent the structural basis of glutamatergic synaptic plasticity [6]. Alterations in spine number and shape are associated with cognitive and developmental dysfunction in various neurological disorders [7]. The possible role of dendritic spines as a cellular substrate for acute and possibly sustained anesthetic effects on the plasticity of mature synapses is an area of intense interest.

Previous studies have shown that anesthetic-induced toxicity and synaptic effects in hippocampal and cortical cultures or slices depend on the neurodevelopmental stage. In early development prior to spine formation, exposure to anesthetics reduces subsequent dendritic spine and filopodial density [8]–[10]. In contrast, during peak synaptogenesis, anesthetic exposure increases dendritic spine density [11], [12]. Intravital imaging of young adult mouse cortical neurons showed that isoflurane had no effects on spine formation or elimination but transiently reduced filopodial elimination [13]. These observations suggest that vulnerability of spines to anesthetics depends on the developmental stage and type of the neuron. However, little research has focused on established dendritic spines, particularly in the hippocampus, an area critical for synaptic plasticity.

General anesthetics potently block hippocampus-dependent memory formation and consolidation, both acutely [14], [15] and chronically [16]–[18], and following exposure during critical periods of neurodevelopment [19], [20]. Hippocampal long-term potentiation (LTP), a neurophysiological correlate of memory, is also blocked by anesthetics [16], [21]. LTP is critically dependent on excitatory synaptic transmission through hippocampal dendritic spines, which are stabilized during memory consolidation [22]. Spine stability is directly regulated by dynamic changes in the actin cytoskeleton. F-actin polymerization increases during hippocampal LTP, and inhibition of actin dynamics leads to attenuation of LTP [23v26]. Mutation or deletion of proteins that regulate actin dynamics are associated with abnormal spine structure, impaired LTP and LTD, and learning and memory deficits [7], [27], [28]. Moreover, the actin cytoskeleton is critical to synaptic transmission and the long-term cellular modifications necessary for learning and memory [29]–[31]. This leads to the concept that amnestic and perhaps sustained neurocognitive effects of anesthetics could involve dendritic spine destabilization. We therefore tested the hypothesis that the representative volatile anesthetic isoflurane destabilizes hippocampal dendritic spines.

## Experimental Methods

### Ethics statement

This study was performed in strict accordance with the recommendations in the Guide for the Care and Use of Laboratory Animals of the National Institutes of Health. The Institutional Animal Care and Use Committee (IACUC) of the University of California San Diego specifically approved this study under protocol #S0729. All of the animals were handled according to this approved protocol. All surgical procedures were terminal and anesthesia with isoflurane was used to prevent animal suffering.

### Hippocampal neuron culture and transfection

Rat hippocampal cells (neurons and glia) were cultured according to Calabrese & Halpain [32]. Briefly, whole hippocampi were dissected from embryonic day 18 Sprague Dawley rats, and the cells dissociated, cultured on glass coverslips (Carolina Biological, Burlington, NC) in 24-well plates (BD Biosciences, San Jose, CA) at a density of 300 cells/mm^2^, and maintained in Neurobasal medium (GIBCO, Grand Island, NY) supplemented with SM1 (Stem Cell Technologies, Vancouver, Canada) and 0.5 mM L-glutamine (Sigma-Aldrich, St. Louis, MO). Cultures were transfected at 21 days in vitro (DIV) using calcium phosphate precipitation with 4–6 µg pEGFP-N1 (Clontech, Mountain View, CA) according to Kohrmann et al. [33] to allow visualization of dendritic morphology. Cells were incubated with the transfection mixture for 2.5 h in 95% air/5% CO_2_ at 37°C, washed twice with pre-warmed HBS solution (in mM: 135 NaCl, 4 KCl, 1 Na_2_HPO_2_, 2 CaCl_2_, 1 MgCl_2_, 10 glucose, and 20 HEPES [pH 7.35]), and replaced with Neurobasal medium. Cells were fixed or used for live cell-imaging experiments 24 to 48 h post-transfection. Only excitatory neurons visually identified by confocal microscopy were analyzed. Inhibitory neurons, which represented <8% of neurons, were identified by their morphology (larger soma, few or no spines) and excluded from analysis.

### Drug treatments

Coverslips were placed in a Plexiglass chamber (0.5 L) within an incubator at 37°C and exposed to 2 vol% isoflurane (∼1.5 MAC in rat [34]), a clinically relevant concentration [35], delivered by a calibrated vaporizer in 95% air/5% CO_2_ at a flow of 2 L/min (for a gas equilibration time constant of 15 sec), or to 95% air/5% CO_2_ alone as a control. Isoflurane is highly lipophilic and rapidly crosses the cell membrane to equilibrate in the cell. In some experiments, neurons were pretreated with 0.1 µM cytochalasin D (Sigma-Aldrich, St. Louis, MO) or 0.1 µM jasplakinolide (EMD Millipore, Billerica, MA) for 2 min prior to isoflurane exposure.

### Phalloidin staining

Control or isoflurane-treated hippocampal cell cultures were fixed with 3.7% formaldehyde in phosphate-buffered saline (PBS) plus 120 mM sucrose for 20 min at 37°C. Fixed cells were rinsed with PBS and permeabilized with 0.2% Triton X-100 in PBS for 4 min at room temperature, then blocked for 30 min with 2% bovine serum albumin (BSA) in PBS. Phalloidin conjugated to Alexa fluor 568 (Invitrogen, Grand Island, NY) diluted 1∶500 was added for 15 min at room temperature. Following rinsing, slides were mounted for imaging.

### Image acquisition and quantitative analysis

**Figure 1:**
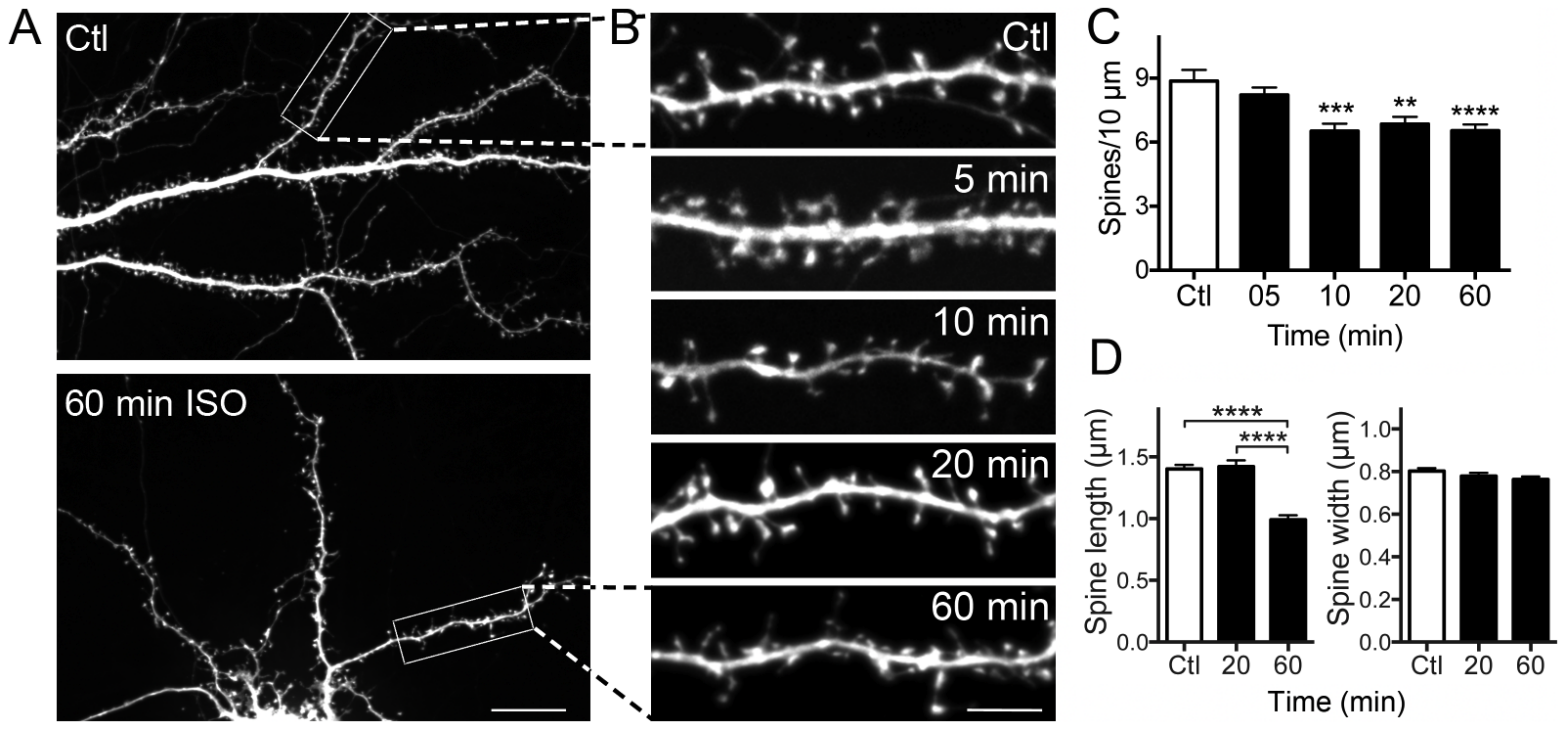
Isoflurane reduces dendritic spine density and alters spine morphology. Hippocampal neuron cultures transfected with eGFP as a cell volume indicator were exposed to 95% air/5% CO_2_ (Ctl) or 2 vol% isoflurane in 95% air/5% CO_2_ for 60 min at 37°C. A-representative full image showing the dendritic arbor of a control neuron and of a neuron treated with isoflurane for 60 min; B-representative images of single ROIs at various times of exposure to isoflurane. Spine number was counted manually at the indicated time points. Isoflurane significantly reduced spine density (C) by one-way ANOVA with Dunnett's post hoc test (**p<0.01; ***p<0.001; ****p<0.0001). Exposure to isoflurane reduced mean spine length (D-left panel) with no significant change in spine width (D-right panel) by one-way ANOVA with Holm-Sidak post hoc test (****p<0.0001). Data are mean ± SEM; n = 30 to 40 dendritic ROIs per experimental group; n = 600 to 1260 dendritic spines for spine density, spine length and spine head width per experimental group. Scale bar in 1A = 10 µm, in 1B = 5 µm.

Fluorescence images were collected using a 60x 1.4 (NA) Plan APO oil immersion objective (Olympus, Tokyo, Japan) and CSU-X1 spinning disk confocal system (Yokogawa, Japan) mounted onto an Olympus IX70 microscope. Samples were excited using a laser launch (Solamere, Salt Lake City, UT) equipped with a 50 mW solid state laser (491 nm) and fluorescence emission was selected through the 525/50 band-pass filter. A series of images was acquired in the z dimension at optical slice thickness of 0.2 to 0.4 µm using a Cool SNAP HQ2 camera (Photometrics, Tucson, AZ). Images were acquired using MetaMorph imaging software (Molecular Devices, Center Valley, PA), and morphometric measurements were analyzed with ImageJ (NIH, Bethesda, MD). Experimental conditions were blinded for analysis. For spine density measurements, three dendrite segments or regions of interest (ROI) of fixed length and width were selected per neuron (Figure 1A). Each ROI contained 15v35 spines. To avoid potential bias associated with differing spine densities along proximal versus distal dendritic locations, proximal and distal regions from a random subset of dendrites were selected. ROIs were selected from 2 proximal and 1 distal region relative to the soma, alternating with 2 distal and 1 proximal region for the next neuron and so on. For spine length and width quantification, length was determined as the distance between the base and top of the spine head, and width was measured across the thickest portion of the spine head. For F-actin concentration, spines were manually outlined and the area within the outline calculated using eGFP fluorescence. The fluorescence intensity of fluorescently labelled phalloidin was thresholded separately, and relative actin concentration was quantified by dividing the thresholded phalloidin fluorescence intensity signal by spine area using ImageJ. From 600 to 1260 spines were analyzed for each experimental group.

### Time-lapse imaging and analysis

Cells were cultured and transfected with eGFP as above on Mat-tek 24-well chambered cover glass (MatTek Corporation, Ashland, MA). Fluorescence emission from transfected neurons was imaged using a 60x 1.4 NA Plan APO oil immersion objective and a CSU-X1 spinning disk confocal system (Yokogawa, Japan) mounted on a Nikon Ti-E microscope with Perfect Focus System (Nikon, Melville, NY) and equipped with a temperature controlled chamber at 37°C ventilated with 95% air/5% CO_2_ (Solent Scientific, Portsmouth, UK). Isoflurane was applied at 2 vol% in 95% air/5% CO_2_ as above. Fluorescence excitation was with a laser launch (Solamere) equipped with a 50 mW solid-state laser (561 nm), and fluorescence emission was selected through a 525/50 band-pass filter. Z-stack images were acquired at 0.2 µm steps with an iXon X3 Du897 EM-CCD camera (Andor Technology, South Windsor, CT). Image stacks were acquired every 5 min for 60 min. A macro written for ImageJ was used to create a mask around the entire neuron using eGFP fluorescence. Using this mask, the dendritic arbor (soma and shaft only) was manually identified and subtracted, leaving only fluorescence of the spine area. Overall spine area was calculated automatically using ImageJ, averaged over each neuron [36], and normalized to initial area from the start of image collection. From 1500 to 1800 spines were analyzed for each experimental group. For the individual spine area analysis, single spines within one ROI were manually outlined using the eGFP signal. The area of individual spines was measured at time points 0, 20 and 60 min and normalized to time = 0 min. Area was categorized into three groups as either increased >10%, decreased >10%, or no change. From 1200 to 1500 spines were analyzed for each experimental group.

### Data analysis

Experimental conditions were blinded for all analysis. Statistical calculations (Student t-test or one- or two-way ANOVA) were performed using Prism v. 5.0 (GraphPad, San Diego, CA) with a threshold for significance of p<0.05. Spine density was calculated using 30 to 50 dendritic regions per group. Phalloidin concentration, spine length, head width and area were calculated using 900 to 1800 spines per group. Data are expressed as mean ± SEM; asterisks indicate values significantly different from control groups (Ctl); *p<0.05, **p<0.01, ***p<0.001, ****p<0.0001.

## Results

### Isoflurane reduces dendritic spine density and shrinks remaining spines

We used rat hippocampal neurons cultured for 3 weeks prior to experimental treatments to examine the effects of isoflurane on mature dendritic spines at established synapses [37]. Spine morphology was visualized in fixed cells using eGFP as a cell volume indicator (Fig. 1A,B). Significant spine loss was observed within 10 min following isoflurane exposure to 2 vol% isoflurane (Fig. 1C), a clinically relevant concentration approximately 1.4 times the EC_50_ for general anesthesia in rat [35]. The decrease in spine number stabilized at 10 min and did not progress further with time. The remaining spines showed a cumulative decrease in mean spine length but not spine width at 60 min compared to control and 20 min (Fig. 1D) corresponding to time-dependent changes in spine morphology.

**Figure 2:**
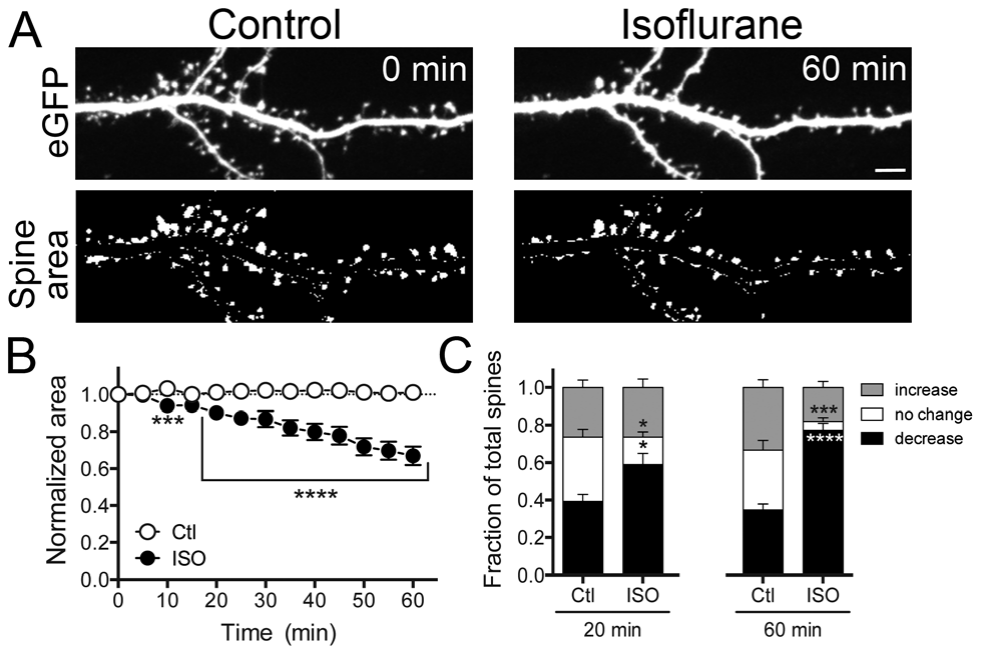
Isoflurane reduces dendritic spine area. Hippocampal neuron cultures transfected with eGFP were exposed to 95% air/5% CO_2_ (Ctl) or 2 vol% isoflurane in 95% air/5% CO_2_ for 60 min at 37°C. Representative images show eGFP fluorescence (A-top panels) and thresholded overall spine area after manual dendrite subtraction (A-bottom panels). A time-dependent decrease in total spine area was observed (B) by two-way ANOVA with Sidak post hoc test (***p<0.001). Changes in individual spine area of >10% (increase) or <10% (decrease), or no change in area were evident at 20 and 60 min (C) by two-way ANOVA with Sidak post hoc test (*p<0.05; ***p<0.001; ****p<0.0001). Data are mean ± SEM; n = 1500 to 1800 dendritic spines per experimental group. Scale bar  = 5 µm.

Time-lapse imaging of live neurons allowed us to observe morphological changes that might have been underestimated using fixed samples. Live cell imaging confirmed that isoflurane induced a time-dependent decrease in overall spine area, resulting in a ∼40% reduction in average spine area after 60 min of continuous exposure compared to time zero (start of image collection) (Fig. 2A,B; Fig. S1). Live cell imaging also allowed us to observe the effects of isoflurane on individual dendritic spines for up to 60 min. In control cultures, spines exhibited characteristic “morphing” behavior, showing continuous, bidirectional changes in spine area, with approximately equal numbers of spines shrinking, growing or not changing relative to time zero. In contrast, isoflurane significantly increased the fraction of shrunken spines at 20 min (59%) and more at 60 min (77%) (Fig. 2C). The fact that we did not observe uniform decreases in size across all spines suggests that individual spines can be relatively sensitive or resistant to isoflurane-induced morphological effects.

### Isoflurane effects on dendritic spines involve the actin cytoskeleton

**Figure 3:**
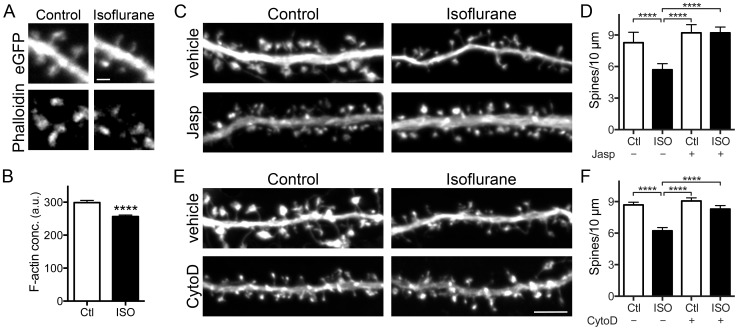
Isoflurane reduces F-actin concentration in spines, and actin targeted drugs prevent isoflurane-induced spine loss. Hippocampal neuron cultures transfected with eGFP were exposed to 95% air/5% CO_2_ (Ctl) or 2 vol% isoflurane in 95% air/5% CO_2_ at 37°C for 20 min and stained with phalloidin-Alexa 568 (A-representative images). Quantification of phalloidin fluorescence intensity shows that isoflurane reduced F-actin concentration (B) by Student unpaired t-test (****p<0.0001). Pretreatment with 0.1 µM jasplakinolide (C-representative images) or 0.1 µM cytochalasin D (E-representative images) for 2 min before exposure to 95% air/5% CO_2_ (Ctl) or 2 vol% isoflurane in 95% air/5% CO_2_ at 37°C for 20 min prevented isoflurane-induced spine loss (D,F) by one-way ANOVA with Tukey's post hoc test (****p<0.0001). Data are mean ± SEM; n = 30 to 40 dendritic ROIs (600–1260 spines) per experimental group for phalloidin staining and actin stabilizing drug treatments. Scale bar  = 1 µm for 3A and 5 µm for 3C,E.

Actin filaments are critical to the development, maintenance, and structural plasticity of dendritic spines [38]. We used fluorescently labeled phalloidin cytochemistry to assess F-actin concentration in spines. In spines remaining following a 20 min exposure to isoflurane, F-actin concentration per spine was significantly reduced (Fig. 3A,B), indicating that reduction of F-actin might underlie isoflurane-induced spine shrinkage and loss. We tested this possibility by pre-incubating cultures for 2 min with either of two compounds that prevent F-actin disassembly. Jasplakinolide, which binds along actin filaments at a site similar to phalloidin and prevents filament disassembly [39], completely blocked isoflurane-induced spine loss (Fig. 3C,D). Cytochalasin D (cytoD), which at the low concentrations used here binds selectively to the barbed (plus) ends of actin filaments and thereby prevents both actin monomer addition and removal from the fast-growing barbed end [40], also completely blocked isoflurane-induced spine loss (Fig. 3E,F). Neither drug alone significantly affected spine number (Fig. 3D,F).

### Isoflurane-induced spine loss and shrinkage are reversible

**Figure 4:**
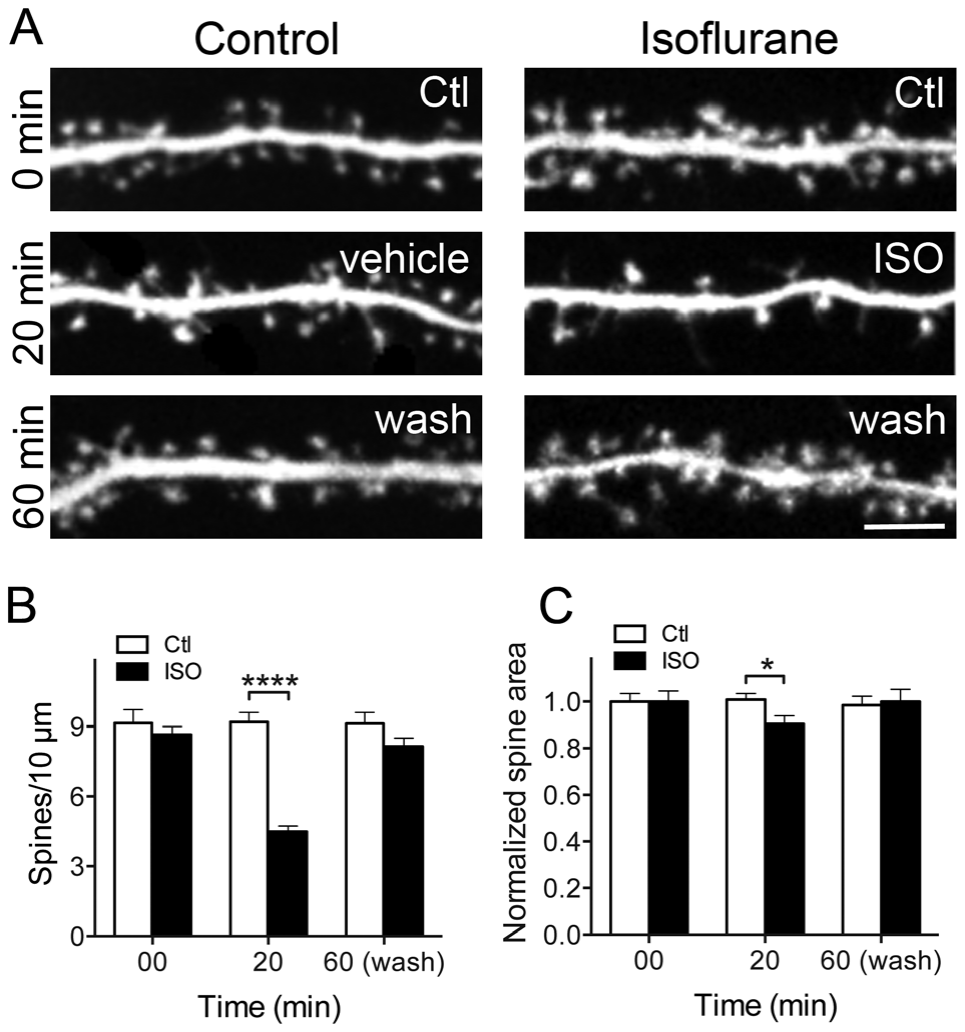
Isoflurane-induced spine loss is reversible. Hippocampal neuron cultures transfected with eGFP were exposed to 95% air/5% CO_2_ (Ctl) or 2 vol% isoflurane in 95% air/5% CO_2_ for 20 min at 37°C. At 20 min, isoflurane exposure was terminated and spine density and area was quantified at different time points (A, only 40 min wash shown). Changes in spine number and area observed after 20 min of isoflurane exposure recovered at 40 min of wash-out (B, C) by two-way ANOVA with Sidak post hoc test (*p<0.05; ****p<0.0001). Data are mean ± SEM; n = 30 to 40 dendritic ROIs per experimental group for spine density; n = 600 to 1800 dendritic spines per experimental group for spine area. Scale bar  = 5 µm.

Dendritic spine stability is essential for normal synaptic function, and permanent changes in spine size and shape can lead to long-term dysfunction. Hippocampal neurons were exposed to isoflurane (2 vol%) for 20 min, and observed following isoflurane wash-out for up to 12 h (Fig. 4A). Spine density (Fig. 4B) and spine area (Fig. 4C) returned to control values within 40 min of isoflurane wash-out following a 20 min exposure. This recovery in spine number and shape was stable up to 12 h (data not shown). These results suggest that isoflurane-induced effects on dendritic spine morphology and number are reversible, and recover upon isoflurane elimination.

## Discussion

We studied the effects of the commonly used volatile anesthetic isoflurane on dendritic spine morphology and stability in mature hippocampal neurons in culture. We found that dendritic spines undergo transient reductions in area and number following exposure to isoflurane, and that actin filament stabilization counteracts this destabilizing effect. Isoflurane effects on actin dynamics provide a plausible mechanism for rapid and transient disruption of synaptic structure, and likely synaptic function as well. This provides a potential structural basis for acute anesthetic actions on hippocampus-dependent memory, and possibly delayed and persistent effects on network function if original connections are not restored.

Dendritic spines are critical to excitatory synaptic transmission in the central nervous system and play important roles in hippocampal synaptic plasticity [6]. General anesthetics alter synaptic plasticity, and have been associated with long-term cognitive dysfunction. Moreover, the hippocampus, a critical center for learning and memory, has been implicated in the amnestic and delayed cognitive effects of anesthetics [14], [41]. We found that a single exposure of synaptically mature hippocampal neurons to a clinically relevant concentration of isoflurane induced reversible decreases in spine size and number within minutes. The rapid reversibility is an important finding arguing against persistent synaptic structural alterations. However, these changes might provide a plausible structural substrate for some of the reported reversible effects of general anesthetics on hippocampal synaptic transmission and plasticity [16], [21], [42]. Previous *in vivo* studies in cerebral cortex also showed no persistent changes in dendritic spine structure with anesthetics [9], [11] consistent with an acute anesthetic effect on synaptic function. Whether these transient alterations in synapse structure we observed are accompanied by enduring changes in synaptic connectivity possibly leading to cognitive dysfunction will require further study. For example, it will be important to identify whether reversible spine shrinkage and loss allows reestablishment of original synaptic connections or whether this reversibility involves formation of new synaptic connections. Spine loss might not be associated with loss of original synaptic connections since spines lost upon excitotoxic glutamate receptor activation reemerge in proximity to their original sites of synaptic contact [43].

The structural changes observed in dendritic spines were mediated by destabilization of actin filaments in the underlying cytoskeleton. The structural destabilization of hippocampal dendritic spines induced by isoflurane correlated with reduced actin filament density detected by phalloidin binding. Importantly, stabilization of actin filaments by two mechanistically distinct actin-modifying drugs prevented spine loss, supporting actin filament destabilization as a downstream mechanism for this isoflurane effect. Cellular actin exists in equilibrium between G-actin monomers and F-actin filaments determined by various actin regulating proteins [44]. Actin filaments have a fast growing barbed end and a slow growing pointed end. During polymerization, G-actin monomers are predominately added to the barbed end unless the filaments are capped. Jasplakinolide binds along the side of actin filaments and thereby prevents polymer disassembly by decreasing the rate of dissociation [45], which likely prevents spine loss because spine structure requires intact actin filaments. CytoD acts by a distinct mechanism involving capping of the barbed end, thereby preventing both G-actin monomer addition to and dissociation from the barbed end [46]. In many cellular structures, actin filaments turnover by disassembly from the pointed ends such that capping the barbed end results in net loss of F-actin within minutes [47]. In dendritic spines however, the pointed ends are thought to be buried within the dense F-actin meshwork [48], and perhaps are present mainly at Arp2/3-mediated filament branch points [49] where they would be protected from monomer dissociation. Thus, pointed ends might turnover quite slowly in spines, such that capping the barbed end with cytoD has the paradoxical effect of stabilizing the spine F-actin meshwork. Our results are consistent with previous reports that actin-stabilizing drugs prevent excitotoxicity-induced spine loss in mature rat hippocampal neurons [50] and attenuate isoflurane-mediated neurotoxicity in immature mouse mixed cortical and hippocampal neurons [51]. Extended incubation with both reagents has been shown to increase spine density [50], [52], so we cannot rule out that they induced increased spine formation, rather than reduced spine loss. However, given the short drug treatment period, the mechanism likely involves reduced spine loss by blocking actin depolymerization. Further studies are required to determine the precise signaling and regulatory mechanisms of isoflurane-induced actin-mediated spine loss.

Previous studies implicated F-actin in anesthetic effects on neurons and astrocytes [8], [51], [53], but have not investigated the stability of established dendritic spines. Matus and colleagues [53] demonstrated that volatile anesthetics reversibly block rapid actin-based spine motility in the spine head (often called “morphing”), but they did not investigate effects on the number and stability of mature dendritic spines. Studies of developing neurons in culture (4–7 DIV) at a stage when dendritic spines have not yet emerged implicated synaptic signaling and actin-dependent pathways in anesthetic-induced neuronal apoptosis [8], [51], but effects on established spine morphology were not reported. Our study suggests that actin-based mechanisms are involved in acute and transient anesthetic destabilization of hippocampal synapses, which are known to be functionally sensitive to volatile anesthetics [3], [4]. Taken together these studies support actin-mediated anesthetic effects on neuronal synaptic structure, function and survival.

General anesthetics depress excitatory transmission both by inhibition of postsynaptic glutamate receptor transmission [54]–[56] and presynaptic inhibition of glutamate release [57]. Actin filaments bind and anchor glutamate receptors to the postsynaptic site [58], [59]. In turn, activation of NMDA receptors can trigger formation of new spines [60]–[62], while AMPA activation stabilizes and maintains existing ones [63]–[65]. Using tetrodotoxin to inhibit electrical activity in mature rat hippocampal neurons, spine stability was required for continuous glutamate signaling [66]. Further studies will determine which actin regulatory proteins mediate isoflurane-induced spine loss and verify if isoflurane-induced changes in spine F-actin are associated with changes in glutamatergic signaling and its downstream targets that determine synapse structure and function.

Dissociated neurons in culture replicate most of the fundamental cellular and molecular aspects of synapse structure and function, and are therefore convenient models systems for studying mechanisms of perturbed function. However, such cultures are ultimately limited in part because they do not reproduce all aspects of intact neuronal networks including their three-dimensional cellular architecture and synaptic connectivity. While our findings are of potential fundamental importance, they should be further verified in more complex physiological and functional systems.

Observations *in vivo* suggest that vulnerability of spines to anesthetics depends on many variables including age, duration of exposure, and brain region [10], [12], [13]. It is not known whether the effects we observed on established hippocampal synapses translate to other brain regions. In immature neurons, both cortical and hippocampal spines showed similar effects of isoflurane during early spine development [8]–[10], [12]. It is important to be cautious when extrapolating the neurodevelopmental stage from rodents to humans and from cell cultures to intact animals. Our finding that not all spines are equally affected by isoflurane suggests that spine-specific properties play a role in the mixed results reported in *in vivo* studies [10], [12], [13]. Given the known pharmacological differences between specific anesthetics involving various molecular targets [1], it will be important to determine both anesthetic agent-specific and neuronal subtype and maturity dependent effects on dendritic spine structure and function.

## Supporting Information

Figure S1 **Isoflurane reduces dendritic spine area.** Hippocampal neuron cultures transfected with eGFP were exposed to 95% air/5% CO_2_ (Ctl) or 2 vol% isoflurane in 95% air/5% CO_2_ for 60 min at 37°C. Representative images show time-lapse of eGFP fluorescence showing a time-dependent decrease in spine area (top panel- Control; bottom panel- Isoflurane). Images taken every 5 min for duration of 60 min.
(GIF)
