# Supplementary figures and images for: Isoflurane Reversibly Destabilizes Hippocampal Dendritic Spines by an Actin-Dependent Mechanism
Source: PLoS One. 2014 Jul 28;9(7):e102978. doi: 10.1371/journal.pone.0102978 (PMC4113311; doi:10.1371/journal.pone.0102978)

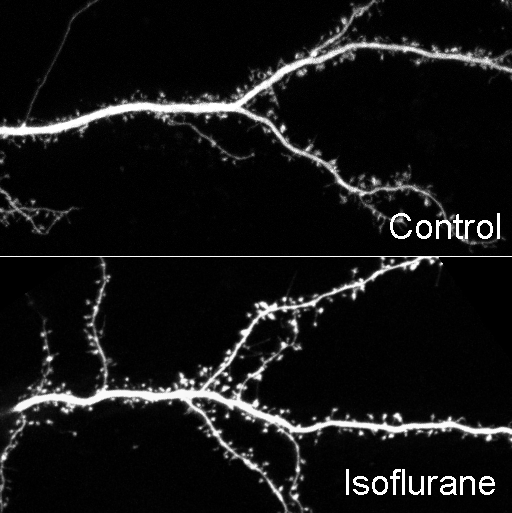

Supplement: Figure S1 — Isoflurane reduces dendritic spine area. Hippocampal neuron cultures transfected with eGFP were exposed to 95% air/5% CO2 (Ctl) or 2 vol% isoflurane in 95% air/5% CO2 for 60 min at 37°C. Representative images show time-lapse of eGFP fluorescence showing a time-dependent decrease in spine area (top panel- Control; bottom panel- Isoflurane). Images taken every 5 min for duration of 60 min. (GIF) [file pone.0102978.s001.gif]
